# Banana Peel Extract-Derived ZnO Nanopowder: Transforming Solar Water Purification for Safer Agri-Food Production

**DOI:** 10.3390/foods13162643

**Published:** 2024-08-22

**Authors:** Dušica Jovanović, Szabolcs Bognár, Vesna Despotović, Nina Finčur, Sandra Jakšić, Predrag Putnik, Cora Deák, Gábor Kozma, Branko Kordić, Daniela Šojić Merkulov

**Affiliations:** 1 Department of Chemistry, Biochemistry and Environmental Protection, University of Novi Sad Faculty of Sciences, Trg Dositeja Obradovića 3, 21000 Novi Sad, Serbia; dusica.jovanovic@dh.uns.ac.rs (D.J.); sabolc.bognar@dh.uns.ac.rs (S.B.); vesna.despotovic@dh.uns.ac.rs (V.D.); nina.fincur@dh.uns.ac.rs (N.F.); branko.kordic@dh.uns.ac.rs (B.K.); 2 Scientific Veterinary Institute “Novi Sad”, Rumenački Put 20, 21000 Novi Sad, Serbia; sandra@niv.ns.ac.rs; 3 Department of Food Technology, University North, Trg Dr. Žarka Dolinara 1, 48000 Koprivnica, Croatia; pputnik@alumni.uconn.edu; 4 Department of Applied and Environmental Chemistry, University of Szeged, Rerrich Béla Square 1, H-6720 Szeged, Hungary; corapravda@gmail.com (C.D.); kozmag@chem.u-szeged.hu (G.K.)

**Keywords:** heterogeneous photocatalytic degradation of organic pollutants, water treatment, green synthesis, eco-friendly synthesis of ZnO nanomaterials, banana peel extract nanoparticles for water purification, Danube River

## Abstract

Pure water scarcity is the most significant emerging challenge of the modern society. Various organics such as pesticides (clomazone, quinmerac), pharmaceuticals (ciprofloxacin, 17α-ethynilestradiol), and mycotoxins (deoxynivalenol) can be found in the aquatic environment. The aim of this study was to fabricate ZnO nanomaterial on the basis of banana peel extract (ZnO/BPE) and investigate its efficiency in the photocatalytic degradation of selected organics under various experimental conditions. Newly synthesized ZnO/BPE nanomaterials were fully characterized by the XRD, FTIR, SEM-EPS, XPS, and BET techniques, which confirmed the successful formation of ZnO nanomaterials. The photocatalytic experiments showed that the optimal catalyst loading of ZnO/BPE was 0.5 mg/cm^3^, while the initial pH did not influence the degradation efficiency. The reusability of the ZnO/BPE nanomaterial was also tested, and minimal activity loss was found after three photocatalytic cycles. The photocatalytic efficiency of pure banana peel extract (BPE) was also studied, and the obtained data showed high removal of ciprofloxacin and 17α-ethynilestradiol. Finally, the influence of water from Danube River was also examined based on the degradation efficiency of selected pollutants. These results showed an enhanced removal of ciprofloxacin in water from the Danube River, while in the case of other pollutants, the treatment was less effective.

## 1. Introduction

The environment faces several severe problems, which bring modern society closer to a true environmental crisis. Environmental pollution is increasing day by day and imposing serious and irreversible damage on the world. Urbanization and technological development endanger the planet’s survival by degrading essential environmental resources, i.e., air, water, and soil, through the release of hazardous wastes [1,2]. Furthermore, the release and transformation of contaminants across terrestrial, aquatic, and atmospheric environments can be caused via climate change. It can impact human health directly through extreme weather events (droughts, flooding, wildfires) and indirectly through ecosystem disruptions [3]. Water pollution is a major concern globally, as all living organisms rely on natural water resources [4]. Despite regulatory norms, millions of tons of toxic materials are discharged into water bodies, causing significant pollution [5].

The global population is expected to be around 10 billion people by 2050, resulting in increased demand for food. Thus, due to the more intense agriculture activities, clean water can be easily contaminated by pesticides. Unfortunately, the applied pesticides negatively affect living organisms. For instance, higher rates of skin and respiratory irritations, as well as conditions like Parkinson’s disease, leukemia, and autism, have been found in residential areas near agricultural lands with continuous pesticide use [6].

The herbicide clomazone (CLO, 2-[(2-chlorophenyl)methyl]-4,4-dimethyl-3-isoxazolidinone) is particularly widely used against species of annual broadleaf weeds and grass. It is highly effective in weed control in the cultivation of soybeans, cotton, rice, sugar cane, corn, tobacco, and a variety of other vegetable crops [7]. However, given its high water solubility (1100 mg/dm^3^) and long half-life dissipation, averaging from 28 to 84 days, it can cause groundwater contamination [8,9].

Quinmerac (QUI, 7-chloro-3-methylquinoline-8-carboxylic acid) is a relatively well soluble in water, hardly degradable synthetic herbicide [10]. Due to the action of precipitation and weak herbicide–soil particle interactions, QUI can move through the soil and thus endanger the quality of groundwater [11]. Substituted quinolinecarboxylic acids are a relatively new class of highly selective auxin herbicides, which effectively control important dicotyledonous weeds in sugar beet, oilseed rape, and wheat [12].

Besides the above-mentioned pesticides, there is another group of pollutants that is poorly recognized. These substances are the active pharmaceutical ingredients (APIs) that, commonly, cannot be completely metabolized in the organism; hence, traces and metabolites are excreted into the aquatic ecosystem. In addition, even though these compounds in waters are present in low concentrations, their persistence poses a threat to aquatic and terrestrial life, and their effects should not be ignored. On the other hand, estimating their long-term effects remains challenging. In the European Union, roughly 3000 different APIs are consumed as medicines, among them analgesics, anti-inflammatories, contraceptives, antibiotics, beta-blockers, lipid regulators, and neuroactive compounds [13].

Ciprofloxacin (CIP, l-cyclopropyl-6-fluoro-4-oxo-7-(piperazin-l-yl)-1,4-dihydroquinoline-3-carboxylic acid) is a widely used antibiotic for treating infections caused by various bacteria. It belongs to the fluoroquinolone class, which has a bioavailability of 69%, has high water solubility, and results in 75% of the ingested dose being excreted into the environment [14]. The use of antibiotics by pharmaceutical manufacturers and hospitals has resulted in wastewater containing significant amounts of these drugs. CIP has been detected in tap water at low concentrations. Moreover, the presence of CIP in wastewater promotes the development of antibiotic-resistant bacteria [15].

17α-ethinylestradiol (EE2, 17α-ethynyl-1,3,5(10)-estratriene-3,17β-diol) is a synthetic estrogen that belongs to a group of endocrine disruptors [16]. Endocrine disruptors are substances that affect the functioning of the endocrine system, leading to harmful health effects. Based on published data, EE2 has been globally detected in various water samples: 1.822 ng/dm^3^ has been detected in marine samples from Australia [17] and 150 ng/dm^3^ in river water samples from Brazil [18], and it is also among the most frequently detected compounds in the EU [19]. The presence of EE2 in nature can lead to harmful health effects, such as reduced sperm quality, genital malformations, or obesity [20].

Mycotoxins are secondary metabolites, typically toxic, produced by the well-known genera *Aspergillus*, *Fusarium*, and *Penicillium*, and they are known to contaminate food and feed. Consuming contaminated crops can lead to mycotoxicosis, causing severe health effects in vertebrates. In addition, humans are also at high risk since mycotoxins can enter the food chain via the direct consumption of infested crops or indirectly via contaminated livestock feed. Existing data indicate a link between mycotoxins and the occurrence of hepatocellular carcinomas [21].

Deoxynivalenol (DON, 3α,7α,15-trihydroxy-12,13-epoxytrichothec-9-en-8-one) is the most widespread mycotoxin from the group of trichothecenes [22]. It is produced by molds of the genus *Fusarium*, which are mycological contaminants of cereals worldwide [23]. The acute toxic effects of DON include gastrointestinal complaints, such as nausea, vomiting, and diarrhea, and there is also evidence of its immunosuppressive effect [24].

As previously mentioned, the clean water shortage represents a severe problem in modern society. Most countries employ different conventional water treatment techniques, such as chemical oxidation, biological treatments, electrochemical degradation, and physico- and physiochemical water treatments. These techniques are ineffective at removing micropollutants, such as pesticides or APIs [25]. Thus, it is necessary to develop powerful and sustainable alternatives. 

Advanced Oxidation Processes (AOPs) are highly effective in the removal of organic pollutants from aqueous environments and have been defined as wastewater treatment techniques since 1987 [26], given that AOPs facilitate the degradation of highly persistent organic substances from water [27]. AOPs are based on the in situ generation of reactive oxygen species (ROS), most commonly hydroxyl radicals (HO^•^); however, there are AOPs that employ sulfate, chloride, or other radicals [28].

Among different AOP techniques, heterogeneous photocatalysis has proven to be an effective tool for removing pesticides and APIs from the aqueous environment [29]. Heterogeneous photocatalysis engages different semiconductors (e.g., ZnO) as photocatalysts and freely accessible sunlight as a source of energy in order to generate wide spectra of ROS, which afterwards attack the present pollutants [30]. 

Photocatalysts are semiconductor materials that become activated upon absorbing photons of radiation [31] Various semiconductor materials are used to remove organic pollutants, but TiO_2_ and ZnO are most commonly applied in heterogeneous photocatalytic reactions due to their excellent characteristics, such as cost-effectiveness, chemical and thermal stability, and safe environmental application, as well as their optical and electrical properties [32]. It should be highlighted that ZnO has increasingly been used instead of TiO_2_ recently, owing to its favorable characteristics, availability, and similar degradation mechanism to that of TiO_2_ [33]. Taking into account the electrical and optical characteristics of ZnO, it can be noted that this semiconductor has a bandgap energy of 3.37 eV and a high binding energy of 60 meV, which explains its high electrochemical stability. Furthermore, ZnO is extremely stable chemically, thermally, and under high-energy radiation [34]. On the other hand, ZnO also possesses some drawbacks, which limit its application as a photocatalyst, such as the necessity of UV radiation for full activation, the rapid recombination of photogenerated electron-hole pairs, the difficult regeneration of ZnO powder from aqueous suspension after treatment, potential aggregation during treatment, and photocorrosion [35].

Besides photocatalysis, indirect photolysis should also be mentioned, since it takes place, for instance, in natural water samples. Indeed, in these samples, various substances are present, such as dissolved organic matter (humic substances) or inorganic anions (nitrate, sulfate, carbonate, chloride), which can act as photosensitizers. Photosensitizers are compounds that absorb photons of radiation and promote the generation of ROS, which then attack the present organic pollutants. However, instead, it is important to note that the presence of photosensitizers can also inhibit the degradation of pollutants, as these substances can also act as scavengers of ROS [36].

Recently, in order to enhance the aforesaid drawbacks of ZnO, great attention has been paid to nanotechnology and its application in various fields, including the synthesis of nanomaterials for photocatalytic purposes [34]. Nanomaterials are a diverse group of resources, and there are several ways to classify them. According to the simplest classification, nanomaterials can be organic and inorganic [37]. There are different synthetic pathways, but the biological approaches are the most important ones, since in these methods, the application of dangerous chemicals is limited; thus, they are believed to be sustainable and harmless to humans and the environment.

Accordingly, great attention has been paid to the application of plant extracts in the synthesis of nanomaterials for engineering various photocatalysts that are crucial for the effective removal of pollutants from the environment [38]. So far, it has been found that plants are the main sources of diverse biomolecules, such as flavonoids, terpenoids, alkaloids, tannins, saccharides, phenols, vitamins, etc. Moreover, they contain various enzymes, amino acids, and proteins, which can also play key roles in different reactions. It is believed that various plant secondary metabolites play crucial roles in the reduction of precursor metal ions, stabilization of obtained nanoparticles, and prevention of their aggregation. Additionally, the keto-enol tautomerism of polyphenols contributes to the release of activated hydrogen, which can participate in the reduction of metal ions that leads to the formation of nanoparticles [39,40,41].

Another important global issue is the fact that the high consumption of food will undoubtedly lead to the high generation of food waste, which, like any other waste, can cause the pollution of the environmental resources. By definition, food waste includes all uneaten food and other waste produced during the preparation of food in households, restaurants, etc. [42]. Non-edible food waste (such as peel, bones, eggshells, etc.) refers to parts of food that are not meant for human consumption under usual circumstances [43]. Bearing that in mind, one inventive way of reducing non-edible food waste is to reuse it during the synthesis of nanomaterials, which can be applied later in water remediation treatment by the photocatalytic process [44,45,46]. The green approach in the synthesis of nanomaterials using extracts derived from food waste is also in harmony with the principles of the circular economy. This model of production and consumption is focused on reducing waste to a minimum through the extension of a product’s life cycle by keeping the original constituents within the economy wherever possible [47]. 

Banana is an edible tropical fruit that belongs to the *Eumusa* section of the genus *Musa*, family *Musaceae*, order *Zingiberales*. Banana fruits are parthenocarpic berries, assembled from the edible pulp that is surrounded by the peel [48]. They have a characteristic medley of bioactive molecules. For instance, in the study by Someya et al. [49], it was found that the total phenolic content in banana peel amounted up to 0.91 g per 100 g dry weight, and the most commonly found phenolic compound was flavanol gallocatechin. Bananas also contain a large amount of biogenic amines, both in peel and pulp, but in particular, the peel is more abundant with catecholamine dopamine (0.08–0.56 g per 100 g of peel) [50]. 

Based on the above-mentioned information, this research focused on the usage of the banana peel, as a type of non-edible food waste, in order to obtain plant extract intended for the preparation of the novel ZnO-based photocatalyst, a green nanomaterial applicable for water purification, while following the fundamental principles of green chemistry and the circular economy with the employment of solar radiation for safer agri-food production. The prepared banana peel-based ZnO material was characterized by employing the following techniques: X-ray powder diffraction (XRD), Fourier transform infrared spectroscopy (FTIR), scanning electron microscopy with energy dispersive X-ray spectroscopy (SEM-EDS), X-ray photoelectron spectroscopy (XPS), and Brunauer–Emmett–Teller (BET) analysis. Furthermore, the photocatalytic activity of the newly synthesized green catalyst was examined in the photocatalytic removal of selected organic pollutants from the aqueous environment using simulated solar irradiation (SSI) by examining various parameters: catalyst loading, initial pH value, and catalyst reusability. Additionally, banana peel extract (BPE) photocatalytic activity was explored. Moreover, the influence of the natural water matrix (water sample from the Danube River) on the photodegradation efficiency of selected organics was assessed. 

## 2. Materials and Methods

### 2.1. Materials, Reagents, and Water Samples

The experiments of photodegradation were conducted using the following components: (i) the herbicide CLO (CAS No. 81777-89-1; 98.8% purity; Sigma–Aldrich, St. Louis, MO, USA) and QUI (CAS No. 90717-03-6; 98.2%; Millipore Sigma Supelco, Darmstadt, Germany), (ii) the APIs CIP (CAS No. 85721-33-1; ≥98% purity; Sigma–Aldrich, St. Louis, MO, USA) and EE2 (CAS No. 57-63-6; ≥98% purity; Sigma–Aldrich, St. Louis, MO, USA), and (iii) the mycotoxin DON (CAS No. 51481-10-8; 98.3% purity; Biopure^TM^, Romer Labs, Tulin, Austria). The major characteristics of investigated pollutants are summarized in Table 1.

The solutions of CLO, QUI, CIP, and EE2 (0.05 mmol/dm^3^) were prepared by dissolving the appropriate mass of the substance in ultrapure water, and it was kept in the dark. Ultrapure water was provided by the Adrona water purification system (LPP Equipment AG, Uster, Switzerland).

The stock solution of the mycotoxin was prepared by dissolving 5.0 mg of the substance in the mixture (19 + 1, *v*/*v*) of ethyl ethanoate (C_4_H_8_O_2_; *M*_r_ = 88.11; CAS No. 141-78-6; G.R. grade; Lach-Ner, s.r.o., Neratovice, Czech Republic) and methanol (CH_4_O; *M*_r_ = 32.04; CAS No. 67-56-1; HPLC gradient grade; Merck, Darmstadt, Germany). The as-prepared stock solution was stored at −18 °C. DON solution, used for the photodegradation experiments, was prepared by evaporating a certain amount of the stock solution and reconstituting the solution in deionized water, so that the final concentration of DON was 5.0 µg/cm^3^ (16.87 µmol/dm^3^). A countertop reverse osmosis system (RO3000-3 and WP4100 SMEG S.P.A., Guastalla, Italy) was used for water purification. 

Additionally, commercial ZnO (99.9%; Sigma–Aldrich, St. Louis, MO, USA; crystallite size of 41.0 ± 0.9 nm, specific pore volume of 0.016 cm^3^/g, and specific surface area of 6.5 m^2^/g [51]) was applied as a photocatalyst in the photocatalytic activity comparison experiments.

**Table 1:** Physicochemical properties of investigated organics [52,53,54,55,56].

Pollutant	Molecular Weight (g/mol)	Chemical Formula	Chemical Structure
CLO	239.70	C_12_H_14_ClNO_2_	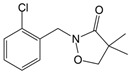
QUI	221.64	C_11_H_8_ClNO_2_	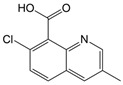
CIP	331.34	C_17_H_18_FN_3_O_3_	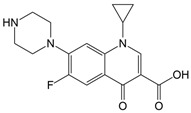
EE2	296.40	C_20_H_24_O_2_	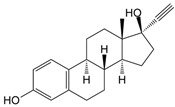
DON	296.32	C_15_H_20_O_6_	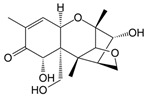

When necessary, pH values were adjusted using 0.1 mol/dm^3^ perchloric acid (HClO_4_; *M*_r_ = 100.46; CAS No. 7601-90-3; 70% (*w*/*w*); 99.99%; Sigma–Aldrich, St. Louis, MO, USA) and 0.1 mol/dm^3^ sodium hydroxide (NaOH; Mr = 39.997; CAS No. 1310-73-2; *p. a.*; MOSS and HeMOSS, Beograd, Serbia). Zinc sulfate heptahydrate (ZnSO_4_ · 7H_2_O; *M*_r_ = 287.56; CAS No. 7446-20-0; 99%; Centrohem, Stara Pazova, Serbia) was used as a ZnO precursor. To prepare the extract of banana (*Musa acuminata*) peel, fresh bananas were purchased from the local market. Acetonitrile (C_2_H_3_N, ACN; *M*_r_ = 41.05; CAS No. 75-05-8; (i) 99.9%, Sigma–Aldrich, St. Louis, MO, USA; and (ii) HPLC gradient grade, Merck, Darmstadt, Germany), phosphoric acid (H_3_PO_4_; *M*_r_ = 97.99; CAS No. 7664-38-2; 85%, *p. a.*, Sigma–Aldrich, St. Louis, MO, USA), and ultrapure water were used as the components of the mobile phase for liquid chromatographic analysis. The surface water sample was collected from the Danube River (Novi Sad, Serbia) in December 2023.

### 2.2. Photocatalyst Synthesis

Synthesis was performed in two consecutive steps. The first step was the preparation of BPE. To remove the surface impurities, banana peels were washed with tap and ultrapure water, dried in the oven at 80 °C, cooled down, and cut into smaller pieces. The pieces of banana peel (220 g) were added to ultrapure water (400 cm^3^) and boiled for 10 min at 80 °C. Boiled pieces of banana peels were separated, blended until smooth, and mixed again with the cooking water. Afterwards, the mixture was filtered through the cloth to obtain the filtrate, i.e., BPE. The second step was concerned with the synthesis of ZnO nanoparticles using BPE. ZnSO_4_ · 7H_2_O (0.08 mol/dm^3^, 1.5 dm^3^) was added to BPE (300 cm^3^), and the pH value of the mixture was set to 12 using NaOH (3.0 mol/dm^3^). The purpose of the BPE addition was to reduce the precursor Zn(II) ions, as well as to stabilize the fabricated nanoparticles and prevent their aggregation. Obtained precipitate was filtered via a Büchner funnel and then washed with ultrapure water to neutral pH. The obtained reaction product was transferred to a petri dish and dried in the oven at 100 °C to a constant mass. The synthesized product, a banana peel extract-based ZnO photocatalyst (ZnO/BPE), was homogenized with mortar and pestle.

### 2.3. Photocatalyst Characterization Techniques

The determination of the ZnO/BPE crystal structure was performed via XRD using a MiniFlex II (Rigaku, Tokyo, Japan) unit outfitted with a Cu *K*α radiation source, with a resolution of 4°/min in the scanning range of 2*θ* = 4–80°. IR spectra were recorded on a Thermo Nicolet iS20 FTIR spectrophotometer (Thermo Fisher Scientific, Waltham, MA, USA) via the KBr pellet method in a range of 4000–400 cm^−1^. The morphological characterization, microstructure, and concentration of elements were determined using APREO C SEM-EDS (Thermo Fisher Scientific, Waltham, MA, USA). Prior to measurement, the samples were sputter-coated with an approximately 5 nm thick layer of gold to prevent charging effects. XPS measurements were conducted using a XPS instrument (Specs-Group, Berlin, Germany) featuring an XR-50 dual-anode X-ray source (Specs-Group, Berlin, Germany) and a Phoibos 150 hemispherical analyzer (Specs-Group, Berlin, Germany). The samples were affixed onto double-sided carbon tape, which was then attached to a stainless-steel sample holder. The Al Kα X-ray source operated at 150 W (14 kV). To counter sample charging, an electron flood gun was employed during the measurements. Survey spectra were acquired with a 40 eV pass energy and a 1 eV step size, while high-resolution spectra were obtained with a 20 eV pass energy and a 0.1 eV step size. The collected high-resolution spectra included C 1*s*, O 1*s*, Zn 2*p*, and Zn LMM (Auger transition). Nitrogen adsorption isotherms were obtained at 77 K using a Nova 2000 surface area analyzer (QuantaChrome, Boynton Beach, FL, USA). Prior to measurement, the samples underwent outgassing at 423 K for 1 h to eliminate any adsorbed contaminants. The specific surface areas were determined using the multipoint BET method based on the six data points of the adsorption isotherms near layer coverage. Pore size distribution curves were derived from the desorption branch of the isotherms using the Barrett–Joyner–Halenda method. The surface fractal dimension (DS) was calculated using the Frenkel–Halsey–Hill method from adsorption data near monolayer coverage.

### 2.4. Measurements of ZnO/BPE and BPE Photocatalytic Activity 

**Figure 1:**
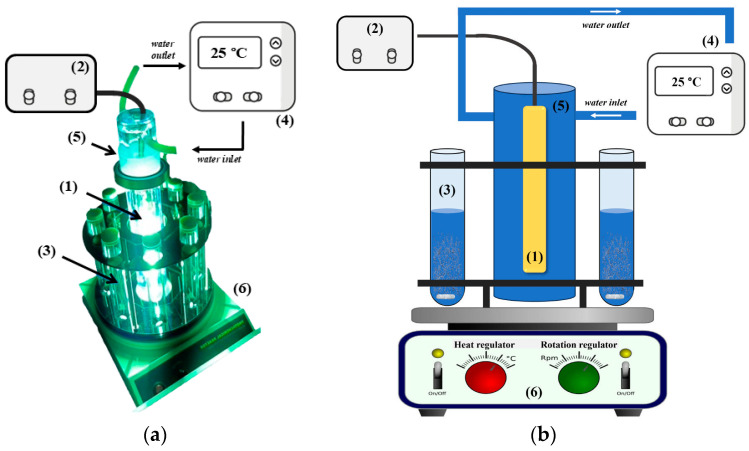
(**a**) Commercial Toption-V batch photoreactor and (**b**) its scheme [57]: xenon lamp (1), lamp controller (2), quartz photochemical cell (3), thermostat (4), quartz cold trap (5), and magnetic stirrer (6).

The experiments of photodegradation were performed in eight identical photochemical cells made of quartz glass (total volume of ca. 100 cm^3^) in a commercial batch photoreactor (Toption-V, Xi’an, China, Figure 1). A xenon lamp (300 W, Toption-V, Xi’an, China) was used as a source of SSI. It was placed in a quartz cold trap, equipped with water-circulating jackets, and connected to a cooler in order to ensure a constant temperature inside the photoreactor. To achieve equal exposure to the irradiation source, photochemical cells were placed in a circle around the xenon lamp. The UV energy fluxes were measured using a portable photo-radiometer (Delta Ohm HD 2102.2, Padova, Italy). The radiometer was equipped with two sensors that correspond to UV (LP 471 UVA, spectral range 315–400 nm) and visible (LP 471 RAD, spectral range 400–1050 nm) radiation. The photon flux for the xenon lamp was 57.52 W/m^2^ for the UVA region and 320.85 W/m^2^ for visible radiation.

Each photodegradation experiment included the addition of pollutant solution (50 cm^3^) and ZnO/BPE or ZnO (except for photolysis) into the photocatalytic cell. Prior to irradiation, in the case of photocatalytic experiments, the suspension was sonicated for 15 min to establish the adsorption–desorption equilibrium on the photocatalyst surface. The experiments concerned with the optimal ZnO/BPE catalyst loading included the addition of (i) 25 mg (*γ* = 0.5 mg/cm^3^), (ii) 50 mg (*γ* = 1.0 mg/cm^3^), or (iii) 100 mg (*γ* = 2.0 mg/cm^3^) of the powder. Each experiment was carried out without pH adjustment, except when the impact of the initial pH value of suspension on the efficiency of CIP removal was investigated in the presence of ZnO/BPE (0.5 mg/cm^3^) under SSI. Photocatalytic activity comparison experiments were conducted under the following process parameters: non-adjusted pH value, initial pollutant (CIP or EE2) concentration = 0.05 mmol/dm^3^, catalyst loading (ZnO/BPE or ZnO) = 0.5 mg/cm^3^, and reaction time = 60 min. 

The photocatalytic activity of BPE was inspected in the removal of CIP and EE2 from ultrapure water. Relative to the initial volume of the pollutant solution (50 cm^3^), different BPE volume fractions (1, 2, and 3%) were added into the photochemical cell. Prior to 60 min of irradiation, the reaction mixture was stirred in the dark for 5 min. 

The river water matrix’s effect on the photodegradation efficiency of selected organic pollutants was studied by dissolving the appropriate mass of the substance in water from the Danube River. Photolytic and photocatalytic experiments were conducted without any pH adjustment, utilizing SSI. ZnO/BPE catalyst loading in these photocatalytic experiments was 0.5 mg/cm^3^, and suspension was sonicated for 15 min prior to irradiation. 

After selected phototreatment intervals (0, 5, 10, 30, 45, and 60 min), degradation samples of selected pollutants were taken and filtered through Millex-GV (0.22 µm, Merck Millipore, Darmstadt, Germany) or nylon (0.22 µm, Amtast, Lakeland, FL, USA) membrane filters to remove the catalyst particles or any other unwanted particulates. The samples were then used in the investigation of photodegradation efficiency.

### 2.5. Photocatalyst Reutilization 

The reutilization study of ZnO/BPE (0.5 mg/cm^3^) was examined in three consecutive runs, each lasting 60 min, in the photocatalytic removal of CIP (0.05 mmol/dm^3^) from ultrapure water (non-adjusted pH value), utilizing SSI. After each run, the suspension was kept overnight in the dark to achieve the precipitation of the ZnO/BPE nanoparticles. After the removal of the supernatant, the photocatalyst was dried in the oven for 2 h at 60 °C. The powder was then used in the photocatalytic degradation experiment of the fresh amount of CIP solution under identical experimental conditions. 

Additionally, the photocatalytic activity of ZnO/BPE material, after storage for six months, was tested in the 60 min degradation of CIP (0.05 mmol/dm^3^) under SSI with 0.5 mg/cm^3^ catalyst loading.

### 2.6. Analytical Procedures

The removal efficiency levels of CLO, QUI, CIP, and EE2 were monitored using a liquid chromatograph with a diode array and fluorescence detector (UFLC-DAD/RF, Shimadzu Nexera, Tokyo, Japan) equipped with a nonpolar Eclipse XDB-C18 column (150 mm × 4.6 mm i.d., particle size 5 μm). The binary mobile phase was composed of ACN and a 0.1% aqueous solution of H_3_PO_4_. Chromatographic analysis was performed in isocratic elution mode under the conditions shown in Table 2.

The degradation efficiency of DON was monitored by a high-pressure liquid chromatograph with a diode array detector (Dionex UltiMate 3000 Series, Thermo Scientific, Germering, Germany) with a Hypersil Aqua GOLD column (150 mm × 3 mm i.d., particle size 3 μm). The chromatographic conditions were as follows: 10:90 (ACN:H_2_O, *v*/*v*), a flow rate of 1.0 cm^3^/min, an injection volume of 50 µL, a column temperature of 30 °C, and an *λ*_max_ of 220 nm.

The pH values were measured using a combined glass electrode (pH-Electrode SenTix 20, WTW; Thermo Fisher Scientific, Waltham, MA, USA) connected to the pH-meter (pH/Cond 340i, WTW).

## 3. Results and Discussion

### 3.1. Characterization of ZnO/BPE

#### 3.1.1. X-ray Powder Diffraction

**Figure 2:**
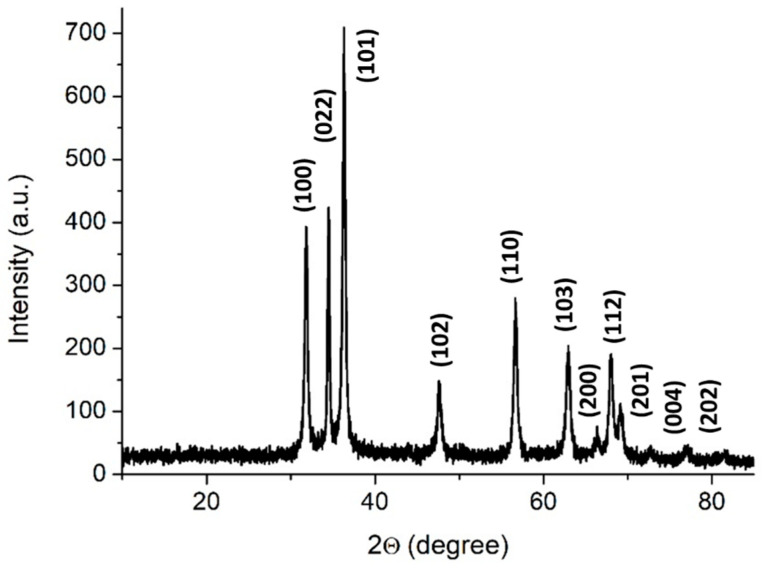
XRD diffractions of ZnO/BPE.

The XRD pattern of ZnO typically shows peaks corresponding to the wurtzite hexagonal phase, which is the most stable and commonly observed phase of ZnO. The main characteristic peaks appear at 2θ values of around 31.7°, 34.4°, and 36.3°, which correspond to the (100), (022), and (101) crystal planes, respectively [58]. These peaks confirm the hexagonal wurtzite structure. The presence of sharp and well-defined peaks without any additional peaks indicates the high phase purity of the ZnO sample. Any secondary phases or impurities would manifest as extra peaks or significant deviations in the expected peak positions. The absence of such anomalies in the XRD pattern confirms that the ZnO sample is phase-pure. The slightly weak intensities are caused by the small size of the ZnO particles, which is also supported by the SEM results. As shown on Figure 2, these diffraction lines were identified in the newly prepared ZnO/BPE.

#### 3.1.2. Fourier Transform Infrared Spectroscopy

**Figure 3:**
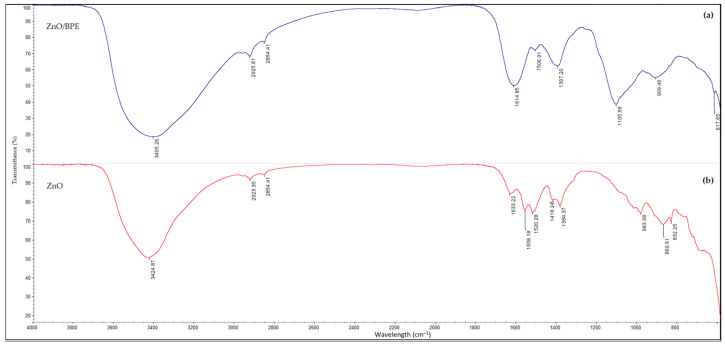
FTIR spectra of (**a**) ZnO/BPE and (**b**) ZnO.

To recognize various functional groups’ newly synthesized ZnO/BPE, the material was characterized using FTIR. The spectrum was compared to commercial ZnO, and the results are shown in Figure 3. Indeed, the absorption peaks within the 1650–800 cm^−1^ range are associated with the organic compounds present in the sample. The absorption peak at 3405.39 cm^−1^ indicates the presence of carboxyl (COOH) and hydroxyl (OH) groups. The band at 1614.85 cm^−1^ originates from the C=C stretching vibrations overlapping with the C=O vibrations originating from the conjugated systems and amide groups, respectively. The complex peak at 1105.68 cm^−1^ can be attributed to the C–O stretch vibration anti-symmetrically coupled to the C–C stretch originating from the alcohol groups. At the same time, the band at 1105.68 cm^−1^ also indicates the presence of phenolic groups, alcohols, and aliphatic amines. The peaks in the range of 900–500 cm^−1^ are attributed to the metal–oxygen groups. The presence of these functional groups is commonly found in biomolecules present in plant extracts [38]. 

#### 3.1.3. Scanning Electron Microscopy with Energy Dispersive X-ray Spectroscopy 

**Figure 4:**
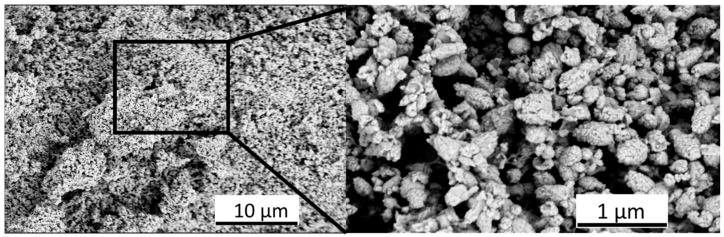
SEM micrographs of ZnO/BPE particles.

Furthermore, based on the SEM-EDS results (Figure 4), it can be stated that the ZnO/BPE particles took an irregular circular shape with smooth edges.

The EDS results have proven that the main components of the sample are Zn and O atoms. However, besides these elements, the ZnO/BPE powder also contains C, Si, and P (Table 3). The present C atoms probably originate from the biomolecules present in the BPE [38]. As expected, the highest intensity signal was given by Zn and O atoms, and their ratio gives a value close to one (Zn:O = 48.45:51.55).

#### 3.1.4. X-ray Photoelectron Spectroscopy

**Figure 5:**
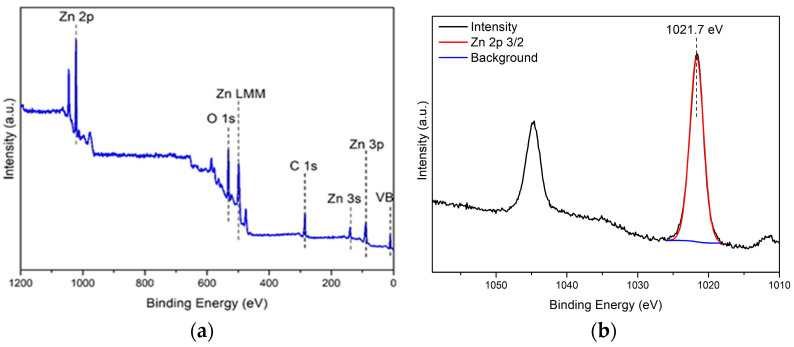
XPS of ZnO/BPE material: (**a**) survey spectrum of ZnO/BPE; (**b**) high-resolution spectrum of Zn 2*p*.

The XPS results (Figure 5) point out that no contaminants were detected in the analyzed ZnO/BPE powder. All high-resolution spectra were background-corrected with a Shirley background. Peaks were fit with a Gaussian–Lorentzian product function where the Lorentzian contribution was 30%. The aliphatic component of the C 1*s* spectrum region 284.8 eV was chosen as an inner reference. The Zn 2*p* 3/2 peak was 1021.7 eV, which indicated that Zn was present as ZnO. This is supported by the binding energy of the Zn LMM Auger peak 988.3 eV. The database of the National institute of standards and technology lists the ZnO LMM Auger peak between 988.1 and 988.5 eV kinetic energy [59]. The sample only contains adventitious carbon (C 1*s*). The O 1*s* component peaks are fit with two component peaks, corresponding to lattice (530.3 eV) and defective oxide (531.7 eV). The defective oxide peak cannot be further split reliably; thus, it also contains the hydrated oxide and hydroxide species. The ratio of the peaks, based on peak areas, is as follows: O_lattice_ = 10.01%, O_defect_ = 89.99%.

#### 3.1.5. Brunauer–Emmett–Teller Analysis

BET measurements (Table 4) revealed that the specific surface area of the newly synthesized ZnO/BPE equals 15.94 m^2^/g. This value is in great agreement with the literature data of the specific surface area of the crystalline ZnO films, which are up to 11.80 m^2^/g. However, it should be taken into account that ZnO materials obtained from various precursor anions show significant differences in BET surface areas (11–85 m^2^/g) [60].

### 3.2. Removal of Organic Pollutants from Ultrapure Water

#### 3.2.1. ZnO/BPE Photocatalyst Loading

It is well known that for every substance, there is an optimal amount of photocatalyst that should be added in an attempt to prevent possible drawbacks caused by higher loadings. However, when this optimal value is surpassed, the decrease in the process efficiency can be observed. For instance, excessive photocatalyst loadings can cause the increase in the suspension opacity and low penetration of the photon flux in the photochemical cell. Simultaneously, at higher photocatalyst loadings, the efficiency can also decrease due to the agglomeration of the photocatalyst particles [61,62]. 

**Figure 6:**
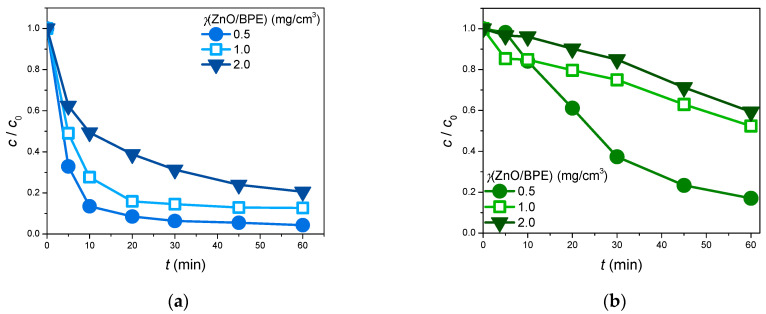
The efficiency of pollutant (0.05 mmol/dm^3^) removal in the presence of different ZnO/BPE loadings under SSI: (**a**) CIP; (**b**) EE2.

Two model compounds, CIP and EE2, were selected to examine the effect of the photocatalyst loading on the pollutant removal efficiency. The obtained results (Figure 6) revealed that the greatest removals of CIP (95.7%) and EE2 (83.0%) were achieved in the system with 0.5 mg/cm^3^ of photocatalyst. Therefore, this value was selected for further experiments. Furthermore, it should be emphasized that the process of photocatalytic degradation had different courses and removal efficiency levels for the two studied pollutants. Indeed, in the case of 0.5 mg/cm^3^ ZnO/BPE loading, 60 min of phototreatment led to the 83.0% removal of EE2, whilst only 10 min of irradiation was enough to reach even higher photocatalytic removal efficiency of CIP (86.7%). Next, when the ZnO/BPE catalyst loading was 1.0 mg/cm^3^, an even shorter irradiation interval (5 min) brought forth a higher removal efficiency for CIP (51.0%) in comparison with 60 min for the EE2 photocatalytic degradation process (47.6%). And, lastly, when it comes to the highest catalyst loading of ZnO/BPE (2.0 mg/cm^3^), comparable removal efficiencies of CIP and EE2 were reached after different time intervals. Indeed, 37.6% of CIP was removed after 5 min of irradiation, whereas EE2 degradation efficiency of 40.7% was achieved after 60 min of photocatalytic treatment. These findings indicate that between the two studied model pollutants, CIP is more prone to removal.

Additionally, the photocatalytic activity of the newly synthesized ZnO/BPE catalyst was compared to that of ZnO, which is a commercially available and commonly used photocatalyst. The results, presented in Table 5 and obtained after 60 min of the process under SSI in ultrapure water as a matrix of choice, revealed that the newly synthesized material contributed to a higher degradation degree of CIP, whilst it caused somewhat lower, but still satisfying, degradation efficiency of EE2 than the commercial ZnO. The greater degradation activity of the ZnO/BPE catalyst could be adequately explained by the results of the BET analysis. Indeed, BET measurements revealed a specific surface area of 14.99 m^2^/g for the newly synthesized nanomaterial. On the contrary, in the study by Finčur et al. [51], the specific surface area of commercial ZnO was found to be about 6.5 m^2^/g. Thus, the results indicate that in the case of the ZnO/BPE catalyst, there is a greater area available for reaction, which can result in improved degradation efficiency. 

#### 3.2.2. BPE Catalytic Activity

**Figure 7:**
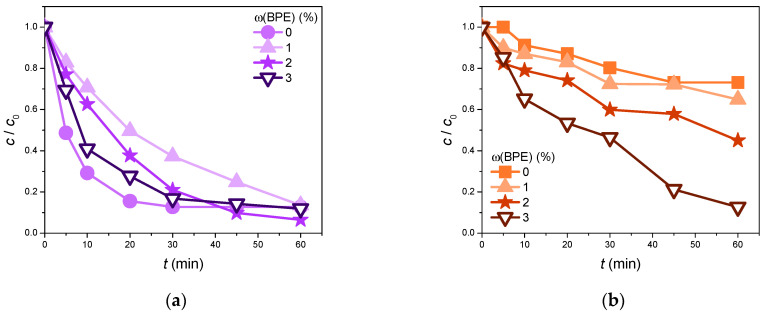
The efficiency of pollutant (0.05 mmol/dm^3^) removal in the presence of different BPE volume fractions under SSI: (**a**) CIP; (**b**) EE2.

To observe whether the BPE alone possesses any catalytic activity, relative to the initial volume of pollutant solution, three distinctive volume fractions of the BPE were studied in the removal of CIP and EE2 from ultrapure water as the aqueous medium. The obtained results were compared to the direct photolysis of CIP and EE2 (ω = 0%) and are presented in Figure 7. It can be seen that the BPE expressed a catalytic activity that is far from negligible. In the case of CIP (Figure 7a), all of the investigated systems ultimately had similar CIP removal efficiencies (86.4, 93.5, and 88.1% in sequence) after 60 min of irradiation. Systems with BPE volume fractions of 1 and 3% displayed CIP removal efficiencies comparable to that of direct photolysis (87.3%). However, the system with 2% BPE proved to be the most effective, as it was more efficient than direct photolysis in removing CIP. Observing the results obtained in the case of EE2 (Figure 7b), it can be noted that the photocatalytic efficiency was higher in the presence of BPE compared to direct photolysis. The increased volume fraction of BPE led to the improved photodegradation efficiency of EE2. Indeed, only 26.8% of EE2 was removed by employing direct photolysis, while 35.1, 55.0, and 87.4% of EE2 was degraded in the system with BPE volume fractions of 1, 2, and 3%, after 60 min of SSI. 

The photocatalytic activity of the BPE can be explained by the fact that plant extracts are rich in various biomolecules, such as terpenoids, polyphenols, phenolic acids, alkaloids, and proteins, which can exhibit photolytic activity [39,63]. These findings underscore the potential for further research into the catalytic properties of plant extracts, as this area has not yet been thoroughly studied.

#### 3.2.3. Initial pH Value of ZnO/BPE Suspension

**Figure 8:**
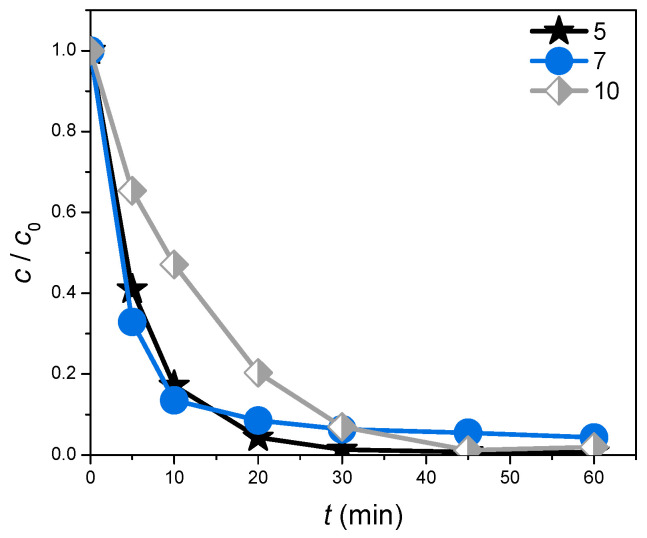
The efficiency of CIP (0.05 mmol/dm^3^) removal in the presence of ZnO/BPE (0.5 mg/cm^3^) under SSI at different initial pH values of the suspension.

Given that the catalyst loading experiments proved that CIP was easier to remove, the experiments concerning the influence of the initial pH value were conducted with CIP as the target pollutant. The impact of the pH was studied at three different levels: 5, 7, and 10. The results (Figure 8) imply that the final removal efficacy of CIP was noteworthy and similar in all three cases (99.3, 95.7, and 98.1%, respectively), meaning that the initial pH value had no significant effect on the removal of CIP. Bearing the economic and environmental aspects of the photocatalytic process in mind, the system without any pH adjustment (pH value = 7) was selected for all further experiments including other substrates as well.

#### 3.2.4. Reutilization Study of ZnO/BPE Photocatalyst 

**Figure 9:**
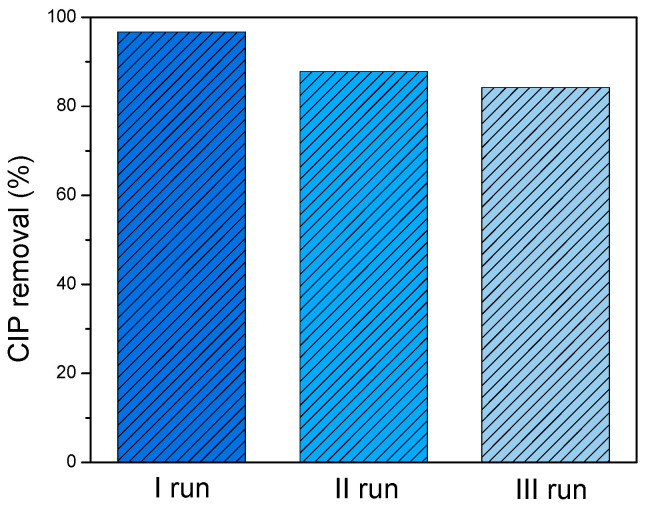
The efficiency of CIP (0.05 mmol/dm^3^) removal in the presence of ZnO/BPE (0.5 mg/cm^3^) after three photocatalytic cycles under SSI.

The important feature of heterogeneous photocatalysis is the ability to reuse the same photocatalyst as much as possible while achieving the same or a somewhat lower pollutant removal efficiency. Photocatalyst reutilization experiments were conducted in three consecutive runs with CIP as the pollutant of choice, in the presence of ZnO/BPE, from the ultrapure water, at the natural pH value, and under SSI. After 60 min of each run, the removal efficiency of CIP was 96.7, 87.8, and 84.2%, respectively. In other words, a minor loss of the photocatalytic potential was observed (Figure 9), but the photocatalyst remained effective even after three photocatalytic cycles, i.e., the material kept its integrity for 3 h. 

**Figure 10:**
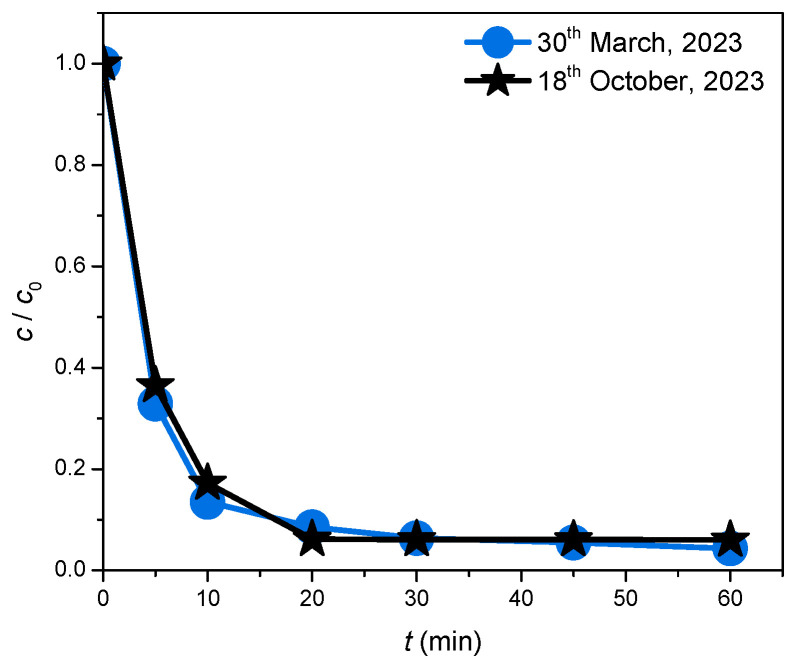
Comparison of CIP (0.05 mmol/dm^3^) removal efficiency in the presence of ZnO/BPE (0.5 mg/cm^3^) and under SSI after storage for a certain period of time.

Additionally, to investigate whether the newly synthesized ZnO/BPE material preserves photocatalytic activity after storage for a certain period of time, one additional experiment was performed with CIP as the selected substrate. As shown in Figure 10, there were minor differences in the course of photocatalytic degradation, but the overall removal efficiency of CIP was nearly identical, meaning that the ZnO/BPE material retained its integrity for more than six months.

### 3.3. The Influence of the Natural Water Matrix on the Photodegradation Efficiency of the Selected Pollutants 

One of the main objectives of the photocatalytic process is the possibility of its application in real life, i.e., in the purification and remediation of natural and wastewaters. 

Therefore, at the non-adjusted pH value and under SSI, the influence of the natural water matrix (water from the Danube River) on the efficiency of the photolytic and photocatalytic degradation of five selected substrates was studied. The physicochemical properties of the water from the Danube River and ultrapure water are displayed in Table 6. 

**Figure 11:**
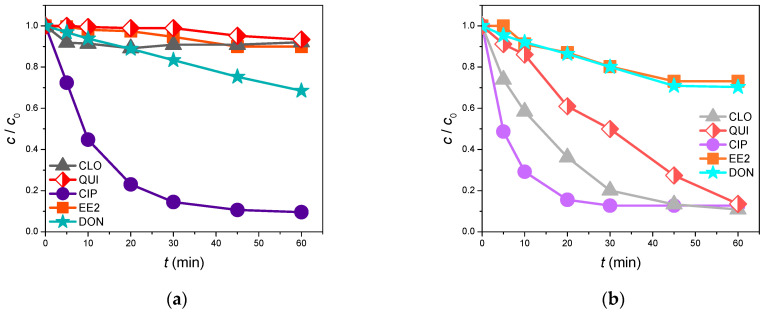
Photolytic removal efficiency of selected organic pollutants (0.05 mmol/dm^3^ for CLO, QUI, CIP, and EE2; 5.0 µg/cm^3^ for DON) under SSI: (**a**) Danube River; (**b**) ultrapure water.

**Figure 12:**
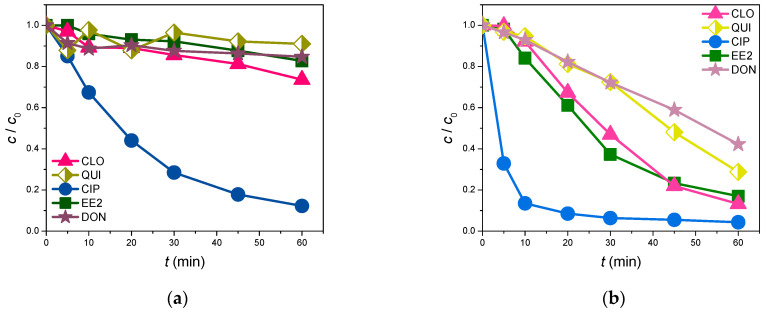
Photocatalytic removal efficiency of selected organic pollutants (0.05 mmol/dm^3^) in the presence of ZnO/BPE (0.5 mg/cm^3^ for CLO, QUI, CIP, and EE2; 5.0 µg/cm^3^ for DON) under SSI: (**a**) Danube River; (**b**) ultrapure water.

Figure 11 and Figure 12 represent how the water matrix influences the photolytic and photocatalytic efficiency in removing the investigated organic pollutants. Indeed, the obtained photolysis results (Figure 11) indicate that CIP and DON behaved almost equivalently in the water from the Danube River and in ultrapure water. Moreover, after 60 min of irradiation, 90.4% of CIP and 31.5% of DON were removed from the Danube River, whereas 87.3% of CIP and 29.7% of DON were removed from ultrapure water. On the other hand, an unexpected effect was observed in the cases of EE2, QUI, and CLO. Here, after 60 min of indirect photolysis, the degradation efficiency decreased more in the water from Danube River than in the ultrapure water. Indeed, in the river water sample, the degradation levels were 10.0, 6.6, and 7.9%, respectively, for EE2, QUI, and CLO, while in ultrapure water, they were 26.9, 86.5, and 89.1%. This slight improvement in the CIP and DON removal efficiency might be attributed to the ions present in the Danube River water that perhaps behaved as photosensitizers, therefore promoting the photodegradation of these pollutants. To this end, in Table 6 it can be seen that in the Danube water, sulfates were detected. Sulfate radical anions can be generated photochemically in the aqueous environment, which then reacts with H_2_O to produce HO^•^, which attacks the present organics [64,65]. Furthermore, the present chloride or bromide anions (Table 6) also influence the degradation efficiency. Even though these anions often separately decrease the photocatalytic activity, there is a possibility of forming chloride/bromide radicals that improve the degradation efficiency [66,67]. Moreover, the presence of nitrate anions (Table 6) can also contribute to the degradation efficiency. Indeed, the nitrate/nitrite anions can be transformed into nitrate/nitrite radicals as a result of hole-induced oxidation. Thus, these newly formed radicals can also attack the organics in water and improve the overall degradation efficiency [68,69].

Upon the initial observation of the obtained results (Figure 12), it can be stated that the Danube River water matrix immensely slowed down the photocatalytic degradation of all studied substrates except for CIP. Specifically, the removal efficiency of CIP reached 87.7% after 60 min of photocatalytic treatment under SSI using the novel ZnO/BPE nano-photocatalyst. This represents an 8% reduction compared to the removal of CIP from ultrapure water. These findings can be explained similarly to those in the case of the photolytic degradation of the selected pollutants. Indeed, the present calcium, potassium, phosphate, magnesium, and sodium cations (Table 6) can decrease the degradation efficiency due to their possible binding to the catalyst surface, trapping the available active sites, thus spatially deactivating the semiconductor. Alternatively, the present anions’ chlorides, bromides, sulfates, and nitrites (Table 6) can cause colloidal instability, greater mass transfer, and dropped surface contact between the selected organics and the photocatalyst [67]. The higher degradation efficiency of CIP can be explained by more favorable interactions between CIP and the applied ZnO/BPE catalyst in the Danube River water. Also, as mentioned earlier, the anions present in river water can act as additional reactive species and enhance the degradation efficiency. This is something that could certainly be a topic of future exploration.

## 4. Conclusions and Outlooks

This research represents a sustainable and innovative approach to water purification, combining the principles of green chemistry and the circular economy with renewable energy to address environmental pollution in the agri-food industry and beyond. Specifically, in this study, a new ZnO catalyst was obtained via green synthesis, using only zinc precursor and banana peel extract as capping agents without calcination, which is a very time- and energy-consuming step, thus fostering principles of sustainability. 

The successful synthesis of ZnO is confirmed by XRD, FTIR and XPS measurements. The XRD pattern shows sharp peaks corresponding to the hexagonal wurtzite structure of ZnO, indicating high phase purity and crystallinity. The FTIR spectra confirmed the presence of organic compounds in the sample. The XPS analysis reveals the presence of Zn^2+^ and O^2−^ ions with no significant impurities, confirming the correct chemical composition. The complementary results from XRD and XPS validate the high purity and quality of the synthesized ZnO. 

Regarding the influence of catalyst loading on the degradation efficiency of CIP and EE2, it can be concluded that 0.5 mg/cm^3^ catalyst loading showed the highest photocatalytic activity, with removal efficiencies of 95% (CIP) and 83% (EE2). The highest photocatalytic activity with ZnO/BPE was achieved in the case of CIP, so the influence of the initial pH on the degradation efficiency of this pollutant was studied. The presented data showed that the initial pH did not have a significant influence on the photocatalytic activity. Furthermore, the reutilization studies showed minimal activity loss after three photocatalytic cycles.

Since an excessive photocatalytic activity of ZnO/BPE was observed, the efficiency of pure BPE was also examined in the degradation of the above-mentioned organics under different BPE volume fractions. The obtained data showed that a volume fraction of 2% BPE was the most suitable for CIP degradation (87.3%), while in the case of EE2, 3% BPE was the most efficient (87.4%). 

The effect of water matrix (Danube River water) was also examined. The degradation efficiency of the selected organics (CLO, QUI, EE2, and DON) was lower in water from the Danube River compared to ultrapure water, with the exception of CIP. Potential interesting avenues of research could include the influence of the ionic content of river water on photodegradation efficiency. 

In the future, further experiments should be carried out in order to gather more information about the photocatalytic activity of pure plant extracts, since it is still a not-exploited field, and special attention should be paid to it. Also, upcoming studies should be even more directed towards the improvement of the reusability of photocatalysts and the possible methods of their recycling, which could be achieved through the immobilization of green photocatalysts on appropriate surfaces, which is the objective of our subsequent research. In addition, other sustainable techniques should be examined and developed for eco-inspired nanomaterial synthesis. Moreover, detailed analysis should be carried out in order to identify the degradation intermediates, including the evaluation of the toxicity of both target organics and photogenerated intermediates in nature, since it is scarce in the field. Taking everything into account, by fulfilling the aforementioned steps and by the additional improvement of heterogeneous photocatalysis, we can completely reduce our presence in nature and develop a powerful technique for the removal of a wide spectrum of organics. 

## Figures and Tables

**Table 2:** Chromatographic conditions applied to analyze the photodegradation samples of herbicides and pharmaceutically active ingredients.

Parameter	CLO	QUI	CIP	EE2
Mobile phase composition (ACN:H_3_PO_4_, *v*/*v*)	60:40	50:50	20:80	80:20
Flow rate (cm^3^/min)	1.0	1.0	0.8	0.7
Injection volume (µL)	20	20	10	10
Column temperature (°C)	25	25	25	40
λ_max_ (nm) ^1^	210	224	279	199
λ_ex_ (nm) ^2^	-	-	280	220
λ_em_ (nm) ^3^	-	-	450	310

^1^ The wavelength of the compound’s maximum absorption. ^2^ The compound’s fluorescence excitation wavelength. ^3^ The compound’s fluorescence emission wavelength.

**Table 3:** Elemental concentrations in ZnO/BPE determined by the EDS technique.

Spectrum	ZnO/BPE
C (wt. %)	28.79
O (wt. %)	36.37
Si (wt. %)	0.18
P (wt. %)	0.16
S (wt. %)	0.34
Zn (wt. %)	34.16

**Table 4:** Textual properties of ZnO/BPE photocatalyst.

Relative Pressure (*p*/*p*_0_)	Total Pore Volume (cm^3^/g)	1/[W((*p*_0_/*p*) − 1)] (1/g)
0.1032	0.4234	217.36
0.1559	0.6456	228.88
0.2031	0.8701	234.33
0.2535	1.2191	222.93
0.3032	1.5941	218.41

**Table 5:** The removal efficiency of CIP and EE2 (0.05 mmol/dm^3^) in the presence of ZnO/BPE or ZnO (0.5 mg/cm^3^) after 60 min of SSI.

	Pollutant	ZnO/BPE	ZnO
**Removal efficiency (%)**	CIP	95.7	94.9
	EE2	83.0	99.8

**Table 6:** The physicochemical properties of the analyzed Danube River water and ultrapure water.

Parameter	Danube River Water	Ultrapure Water
pH	8.10	6.56
Conductivity at 25 °C (μS/cm)	424	4.5
TOC (mg/dm^3^)	2.30	<DL
Fluoride (mg/dm^3^)	<DL	<DL
Chloride (mg/dm^3^)	44.02	<DL
Bromide (mg/dm^3^)	0.080	<DL
Sulfate (mg/dm^3^)	15.52	<DL
Nitrate (mg/dm^3^)	<DL	<DL
Nitrite (mg/dm^3^)	0.024	<DL
Calcium (mg/dm^3^)	0.136	<DL
Potassium (mg/dm^3^)	0.030	<DL
Lithium (mg/dm^3^)	<DL	<DL
Phosphates (mg/dm^3^)	0.202	<DL
Magnesium (mg/dm^3^)	0.078	<DL
Sodium (mg/dm^3^)	0.043	<DL
Ammonium (mg/dm^3^)	0.11	<DL

## Data Availability

The original contributions presented in the study are included in the article, further inquiries can be directed to the corresponding author.
